# Personalized Medicine in Acromegaly: The ACROFAST Study

**DOI:** 10.1210/clinem/dgae444

**Published:** 2024-06-29

**Authors:** Montserrat Marques-Pamies, Joan Gil, Miguel Sampedro-Nuñez, Elena Valassi, Betina Biagetti, Olga Giménez-Palop, Marta Hernández, Silvia Martínez, Cristina Carrato, Rocío Villar-Taibo, Marta Araujo-Castro, Concepción Blanco, Inmaculada Simón-Muela, Andreu Simó-Servat, Gemma Xifra, Federico Vázquez, Isabel Pavón, José Antonio Rosado, Rogelio García-Centeno, Roxana Zavala, Felicia Alexandra Hanzu, Mireia Mora, Anna Aulinas, Nuria Vilarrasa, Soledad Librizzi, María Calatayud, Paz de Miguel, Cristina Alvarez-Escola, Antonio Picó, Isabel Salinas, Carmen Fajardo-Montañana, Rosa Cámara, Ignacio Bernabéu, Mireia Jordà, Susan M Webb, Mónica Marazuela, Manel Puig-Domingo

**Affiliations:** Department of Endocrinology and Nutrition, Hospital Municipal de Badalona, Badalona 08911, Spain; Endocrine Research Unit, Germans Trias i Pujol Research Institute (IGTP), Badalona 08916, Spain; Centro de Investigación Biomédica en Red de Enfermedades Raras (CIBERER, Unidad 747), Instituto de Salud Carlos III (ISCIII), Barcelona 28029, Spain; Department of Endocrinology and Nutrition, La Princesa University Hospital, Madrid 28006, Spain; Endocrine Research Unit, Germans Trias i Pujol Research Institute (IGTP), Badalona 08916, Spain; Centro de Investigación Biomédica en Red de Enfermedades Raras (CIBERER, Unidad 747), Instituto de Salud Carlos III (ISCIII), Barcelona 28029, Spain; Department of Endocrinology and Nutrition, Germans Trias i Pujol University Hospital, Badalona 08916, Spain; Department of Endocrinology and Nutrition, Vall Hebron University Hospital, Barcelona 08035, Spain; Department of Endocrinology and Nutrition, Parc Taulí University Hospital, Sabadell 08208, Spain; Department of Endocrinology and Nutrition, Arnau de Vilanova University Hospital, Lleida 25198, Spain; Endocrine Research Unit, Lleida Institute for Biomedical Research Dr. Pifarré Foundation (IRBLleida), Lleida 25198, Spain; Department Hormonal Laboratory, Germans Trias i Pujol University Hospital, Badalona 08916, Spain; Department of Pathology, Germans Trias i Pujol University Hospital, Badalona 08916, Spain; Department of Endocrinology and Nutrition, Clínico de Santiago University Hospital, Santiago de Compostela 15706, Spain; Department of Endocrinology and Nutrition, Ramón y Cajal University Hospital, Madrid 28034, Spain; Instituto de Investigación Ramón y Cajal (IRYCIS), Madrid 28034, Spain; Department of Endocrinology and Nutrition, Príncipe de Asturias University Hospital, Madrid 28805, Spain; Department of Endocrinology and Nutrition, Joan XXIII University Hospital, Tarragona 43005, Spain; Endocrine Research Unit, Institut d´Investigació Sanitària Pere Virgili (IISPV), Tarragona 43005, Spain; Rovira i Virgili University (URV), Tarragona 43003, Spain; Endocrine Research Unit, Institut d'Investigació Biomèdica de Bellvitge (IDIBELL), Hospitalet de LLobregat 08907, Spain; Department of Endocrinology and Nutrition, Mutua de Terrassa University Hospital, Terrassa 08221, Spain; Department of Endocrinology and Nutrition, Josep Trueta University Hospital, Girona 17007, Spain; Department of Endocrinology and Nutrition, Germans Trias i Pujol University Hospital, Badalona 08916, Spain; Department of Endocrinology and Nutrition, Getafe University Hospital, Madrid 28905, Spain; Department of Endocrinology and Nutrition, Getafe University Hospital, Madrid 28905, Spain; Department of Endocrinology and Nutrition, Gregorio Marañón University Hospital, Madrid 28007, Spain; Department of Endocrinology and Nutrition, Joan XXIII University Hospital, Tarragona 43005, Spain; Department of Endocrinology and Nutrition, Hospital Clinic University Hospital, Barcelona 08036, Spain; Endocrine Research Unit, Institut d’Investigacions Biomèdiques August Pi I Sunyer (IDIBAPS), Barcelona 08036, Spain; Department of Endocrinology and Nutrition, Hospital Clinic University Hospital, Barcelona 08036, Spain; Endocrine Research Unit, Institut d’Investigacions Biomèdiques August Pi I Sunyer (IDIBAPS), Barcelona 08036, Spain; Centro de Investigación Biomédica en Red de Enfermedades Raras (CIBERER, Unidad 747), Instituto de Salud Carlos III (ISCIII), Barcelona 28029, Spain; Department of Endocrinology and Nutrition, Research Center for Pituitary Diseases, Institut de Recerca Sant Pau (IIB-Sant Pau), Hospital Sant Pau, Barcelona 08041, Spain; Endocrine Research Unit, Institut d'Investigació Biomèdica de Bellvitge (IDIBELL), Hospitalet de LLobregat 08907, Spain; Department of Endocrinology and Nutrition, Bellvitge University Hospital, Hospitalet de Llobregat 08907, Spain; Centro de Investigación Biomédica en Red de Diabetes y Enfermedades Metabólicas (CIBERDEM), Instituto de Salud Carlos III (ISCIII), Madrid 28029, Spain; Department of Endocrinology and Nutrition, 12 de Octubre University Hospital, Madrid 28041, Spain; Department of Endocrinology and Nutrition, 12 de Octubre University Hospital, Madrid 28041, Spain; Department of Endocrinology and Nutrition, Clínico San Carlos University Hospital, Madrid 2546, Spain; Department of Endocrinology and Nutrition, La Paz University Hospital, Madrid 28046, Spain; Department of Endocrinology and Nutrition, General University Hospital Dr Balmis, Miguel Hernández University, Alicante 03010, Spain; Endocrine Research Unit, Instituto de Investigación Sanitaria y Biomédica de Alicante (ISABIAL), Alicante 03010, Spain; Department of Endocrinology and Nutrition, Germans Trias i Pujol University Hospital, Badalona 08916, Spain; Department of Endocrinology and Nutrition, La Ribera University Hospital, Valencia 46600, Spain; Department of Endocrinology and Nutrition, La Fe University Hospital, Valencia 46026, Spain; Department of Endocrinology and Nutrition, Clínico de Santiago University Hospital, Santiago de Compostela 15706, Spain; Endocrine Research Unit, Germans Trias i Pujol Research Institute (IGTP), Badalona 08916, Spain; Centro de Investigación Biomédica en Red de Enfermedades Raras (CIBERER, Unidad 747), Instituto de Salud Carlos III (ISCIII), Barcelona 28029, Spain; Department of Endocrinology and Nutrition, Research Center for Pituitary Diseases, Institut de Recerca Sant Pau (IIB-Sant Pau), Hospital Sant Pau, Barcelona 08041, Spain; Departament de Medicina, Universitat Autònoma de Barcelona (UAB), Bellaterra 08193, Spain; Department of Endocrinology and Nutrition, La Princesa University Hospital, Madrid 28006, Spain; Endocrine Research Unit, Germans Trias i Pujol Research Institute (IGTP), Badalona 08916, Spain; Centro de Investigación Biomédica en Red de Enfermedades Raras (CIBERER, Unidad 747), Instituto de Salud Carlos III (ISCIII), Barcelona 28029, Spain; Department of Endocrinology and Nutrition, Germans Trias i Pujol University Hospital, Badalona 08916, Spain; Departament de Medicina, Universitat Autònoma de Barcelona (UAB), Bellaterra 08193, Spain

**Keywords:** acromegaly, medical treatment, personalized therapy, first-generation somatostatin receptor ligands, therapeutic response prediction, clinical trial

## Abstract

**Context:**

Medical treatment of acromegaly is currently performed through a trial-and-error approach using first-generation somatostatin receptor ligands (fgSRLs) as first-line drugs, with an effectiveness of about 50%, and subsequent drugs are indicated through clinical judgment. Some biomarkers can predict fgSRLs response.

**Objective:**

Here we report the results of the ACROFAST study, a clinical trial in which a protocol based on predictive biomarkers of fgSRLs was evaluated.

**Methods:**

This was a prospective trial (21 university hospitals) comparing the effectiveness and time-to-control of 2 treatment protocols during 12 months: (A) a personalized protocol in which the first options were fgSRLs as monotherapy or in combination with pegvisomant, or pegvisomant as monotherapy depending on the short acute octreotide test (sAOT) results, tumor T2 magnetic resonance (MRI) signal or immunostaining for E-cadherin; and (B) a control group with treatment always started by fgSRLs and the other drugs included after demonstrating inadequate control.

**Results:**

Eighty-five patients participated; 45 in the personalized and 40 in the control group. More patients in the personalized protocol achieved hormonal control compared to those in the control group (78% vs 53%, *P* < .05). Survival analysis revealed a hazard ratio for achieving hormonal control adjusted by age and sex of 2.53 (CI, 1.30-4.80). Patients from the personalized arm were controlled in a shorter period of time (*P* = .01).

**Conclusion:**

Personalized medicine is feasible using a relatively simple protocol, and it allows a higher number of patients to achieve control in a shorter period of time.

Medical treatment of acromegaly is currently performed through a trial-and-error approach using first-generation somatostatin receptor ligands (fgSRLs) as first-line drugs, with the possibility of adding cabergoline, pegvisomant, and/or pasireotide upon clinical judgment in case of inadequate response (1, 2). The reported average effectiveness of fgSRLs is around 50% (3-6) and several months of treatment are required to establish the response. Thus, it implies a considerable delay in the control of the acromegaly status when no adequate response to fgSRLs is initially obtained, and subsequent different drugs must be tried.

Some biomarkers have been reported so far that are able to predict response to fgSRLs, including functional, radiological, and molecular markers (7, 8). A low growth hormone (GH) at 2 hours (GH_2h_) after the short acute octreotide test (sAOT) has been associated with a better response to fgSRLs, with a predictive value for IGF1 normalization of about 80% to 90% (9-11). Patients with tumors harboring an hypointense T2 magnetic resonance imaging (MRI) signal more frequently present a complete response to fgSRLs relative to patients with hyperintense or isointense tumors (12-16), with an accuracy of 80% for identifying a GH reduction of > 80% (17). The expression of different molecules at tumor tissue level, such as somatostatin receptor 2 (SSTR2) (18-23), E-cadherin (22-25), as well as Ki-67 labeling index and granulation pattern (23, 26), have also been recognized as good predictors for response to fgSRLs. Moreover, an algorithm including a combination of these biomarkers to individualize medical treatment and improve the effectiveness of its management has already been proposed (27). An adequate control of acromegaly has demonstrated to decrease comorbidities, to reduce mortality rates among these patients (28-31) and to improve patient quality of life (32, 33).

Here we report the results of the ACROFAST study, the first prospective trial that evaluates a personalized medical treatment algorithm based on biomarkers predicting the response to fgSRLs compared to a control group in which standard treatment was used. The personalized treatment arm included first-line fgSRLs, pegvisomant, or their combination according to biomarkers response prediction. The primary outcomes were the frequency of patients achieving hormonal control and the time-to-control using both protocols, with the hypothesis that the personalized protocol would be more efficient for medical therapy of acromegaly.

## Methods

### Study Design

A prospective multicenter trial was set up in 21 tertiary referral centers in Spain: personalized treatment was given in 10 centers and standard treatment in 11 centers.

The study included both recently diagnosed patients who were naïve to medical treatment and postsurgical noncured cases. Evaluation of the hormonal control and the acromegaly comorbidities evolution was performed every 3 months by GH and IGF1 determinations, until control of the disease and a total maximum follow-up period of 12 months. A control MRI was performed every 3 to 6 months to assess tumoral changes. Adverse events and therapeutic compliance were also assessed at every visit. Patients who were non-adherent to the study protocol or who presented adverse effects that prevented achieving maximal doses of assigned medical treatment were excluded from the study.

An external independent committee evaluated the interim trial results for the possibility of one of the arms presenting extremely divergent results, in which case the trial would be required to be stopped. They also evaluated the protocol deviations and adverse events that could influence protocol compliance.

### Patients

From December 2019 to December 2022, participants were prospectively recruited. Inclusion criteria were 18-80 years of age; acromegaly diagnosis as defined by clinical guidelines; signed informed consent and patient's ability to comply with the study protocols. Participants were included at the moment of the diagnosis or if they were not cured 3 months after surgical treatment and were assessed while no medical therapy was given. According to the inclusion criteria, a patient could be included twice: before surgery and after surgery if the patient had not been cured. This situation happened in 3 cases: 2 patients from the personalized group and 1 patient from the control group. Exclusion criteria were medical treatment for acromegaly during the last 3 months, previous radiotherapy, pregnancy, renal failure (estimated glomerular filtration rate [eGFR] < 30 mL/min/1.73 m^2^) and severe liver disease (encephalopathy, ascites, coagulopathy, or hypoalbuminemia).

The study was conducted in accordance with the ethical principles of the Declaration of Helsinki and implemented and reported in accordance with the International Conference on Harmonised Tripartite Guideline for Good Clinical Practice. The study was approved by the Germans Trias i Pujol Hospital Ethics Committee for Clinical Research (Ref.: PI-19-054). The protocol and informed consent forms were also approved by the institutional review board of all the participating centers, independent ethics committee, and/or research ethics board of each study site. All patients provided written informed consent to participate in the study.

### Biomarkers Used for fgSRL Response Prediction

The following response predictor biomarkers to fgSRLs were used in the personalized group to define the specific medical treatment:

#### Short acute octreotide test

At the inclusion of the study, a sAOT was performed in each center. The sAOT consisted of collecting a basal blood sample for GH measurement, followed by the subcutaneous administration of 100 mcg of regular octreotide, and a second blood extraction 2 hours later. The GH_2h_ value was considered equivalent to the GH nadir (GH_nad_) as previously described (11). To evaluate GH suppression, either GH_2h_ or the percentage of GH decrease from baseline (%∇GH) were used. A GH_2h_ cutoff of below 2.7 ng/mL was defined to identify responders to fgSRLs according to previous data from our group (11), in which the aforementioned 2.7 ng/mL value was obtained from extrapolation of the originally described one to the values obtained with the current ultrasensitive GH assays, following the criteria described by Müller et al (34). Thus, if the sAOT GH_2h_ was < 2.7 ng/mL, the patient was considered a responder; if GH_2h_ was > 2.7 ng/mL but the %∇GH was higher than 50%, the patient was classified as intermediate responder, and if it was lower than 50% the patient was classified as a non-responder (Table 1).

**Table 1.** Short acute octreotide test interpretation

GH_2h_ (ng/mL)	%∇GH	Response classification
< 2.7		Responder
> 2.7	Decrease > 50% from baseline	Partial responder
> 2.7	Decrease < 50% from baseline	Non-responder

Determination of growth hormone (GH) 2 hours after the administration of 100 mg of octreotide subcutaneous (GH_2h_). GH decrease 2 hours after the administration of 100 mg of octreotide subcutaneous (%∇GH).

#### Magnetic resonance imaging

MRI was performed at baseline (for newly diagnosed patients and for noncured postoperative patients) to assess tumor size, extrasellar invasiveness, and T2 signal intensity. In case of cavernous sinus invasion, the Knosp classification was used for grading. A control MRI was performed between 3 and 6 months after having initiated medical treatment to evaluate changes in tumor size (highest diameter and volume). Tumor volume was calculated by the Di Chiro and Nelson formula: volume = height × length × width × π/6 (35), which was done by a neuroradiologist from the pituitary multidisciplinary committee in each center. The intensity of the tumor or its remnant was compared to that of normal pituitary tissue. When normal pituitary tissue was not visible, the gray matter of the temporal lobe was used as a comparator (13). The presence of T2 hypointensity was considered a marker of good response to fgSRLs, while T2 iso- or hyperintensity was considered a marker of poor response to fgSRLs.

#### Immunohistochemistry

Formalin-fixed paraffin-embedded tumor samples were cut into 4-µm-thick sections and stained using a fully automated Ventana BenchMark ULTRA stainer (Ventana, Tucson, AZ, USA) according to the manufacturer's instructions. E-cadherin immunohistochemistry was performed after surgery when tumor tissue from operated patients was available, which was possible in 24 out of 30 patients in the personalized treatment arm. Additionally, it was also performed in 12 patients from the standard treatment arm. We used the mouse monoclonal anti-E-cadherin antibody (RRID AB_397580) (Ventana, Tucson, Ariz., USA) purchased as a prediluted antibody, with a concentration of 0.314 µg/dL. E-cadherin was scored in 2 intensities as negative (when the adenoma cells seemed negative at low [×40] and at high [× 200] magnification) and positive (when the adenoma cells were positive at low [×40] or high [× 200] magnification). No differentiation was made between strong and weak positive adenomas because the same fgSRLs response has been described for both (22). The immunohistochemistry studies were centralized in a single center and performed by the pathologist of the pituitary multidisciplinary committee from our center (C.C.).

### Hormonal Determinations

Hormonal measurements included the whole set of pituitary hormones, as well as GH and IGF1 for acromegaly diagnosis. Achievement of normal IGF1 was used to define acromegaly control by local laboratories.

Serum GH was measured at each center by different automated immunoassays, all calibrated against World Health Organization (WHO) International Standard 98/574: Immulite i2000, Siemens Healthineers (RRID:AB_2811291); Liason XL, Diasorin (RRID:AB_3099571); UniCel DxI 800 Access, Beckman Coulter (RRID:AB_2756876) and Cobas 8000, Roche Diagnostics (RRID:AB_2883974). To ensure consistency and comparability of GH measurements obtained from different immunoassays and centers, the results were harmonized according to Müller et al (34) with a linear regression equation for each assay adjusting the GH concentrations of each immunoassay to a reference immunoassay (Immulite i2000). The Passing-Bablok regression equations were for Liason XL: y = 1.272x + 0.023 and for DxI 800: y = 1.387x + 0.356. To harmonize the results of the Roche immunoassay we used the Passing-Bablok regression equation obtained by a method comparison of 51 samples measured by both immunoassays (Immulite i2000 and Cobas 8000). The regression equation obtained was y = 1.089x + 0.082. Through the application of these regression equations, all GH values used in the study were standardized, ensuring uniformity across different immunoassays and centers, to the cutoff values predicting responsiveness (2.7 ng/dL). Serum IGF1 concentrations were also measured in each center by immunoassays calibrated against WHO NISBC 2stIS 02/254: Liason XL, Diasorin (RRID: AB_2928957), Immulite i2000, Siemens Healthineers (RRID:AB_2922766) and ELISA Mediagnost (RRID:AB_2813791). IGF1 concentrations were evaluated as absolute concentrations, and they were calculated as IGF1-SDS for outcomes assessment and inter-center comparability. IGF1-SDS was calculated using the calculator available online from the Spanish Society of Endocrinology and Nutrition website (www.seen.es/portal/calculadoras/sds-igf-1; last accessed November 11, 2023).

### Treatment Algorithms

Medical treatments included in this study were fgSRLs initiated at medium doses (octreotide LAR 20 mg every 4 weeks or lanreotide 90 mg every 4 weeks), pegvisomant with a starting dose of 0.5 mg/kg/week dose and administered on alternate days, and a combination of both at the same doses than in monotherapy (fgSRLs + pegvisomant). For cases with minor elevations of IGF1 (2.5-3 SDS), cabergoline at a dose of 1 mg/week was also considered combined with fgSRLs.

**Figure 1.**
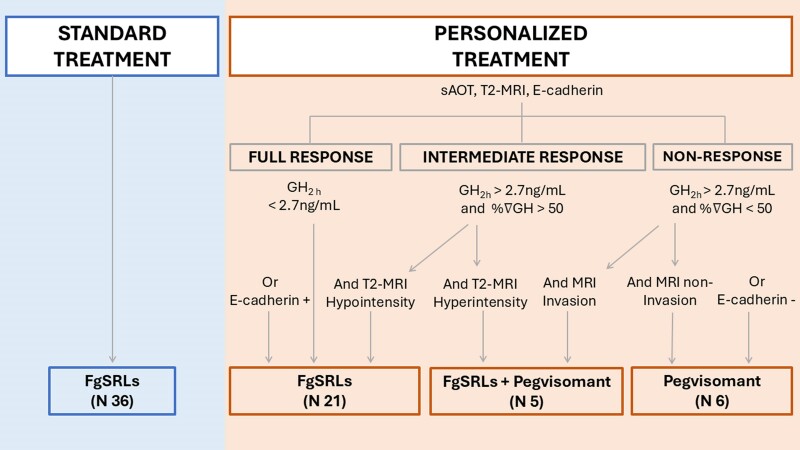
Treatment algorithms. After inclusion, patients were treated according to the standard treatment or a personalized treatment based on the short acute octreotide test (sAOT), T2-MRI intensity, and the expression of E-cadherin. Abbreviations: fgSRLs, first-generation somatostatin receptor ligands; GH, growth hormone; GH_2h_, growth hormone value 2 hours after the short acute octreotide test; MRI, magnetic resonance imaging; PEGV, pegvisomant; %∇GH, percentage GH variation after the short acute octreotide test; (PEGV).

Thus, the 2 treatment algorithms compared in this study were a personalized algorithm and a standard treatment algorithm (shown in Fig. 1 and detailed below).

#### Personalized algorithm

In the personalized algorithm, nonoperated cases were treated with different drugs according to the GH_2h_ and the %∇GH after the sAOT results, the T2 MRI intensity, and the presence of sinus invasion.

For patients with persistent acromegaly recruited after a first surgery, treatment was established according to E-cadherin immunopositivity or immunonegativity expression. By exception, in those postoperative cases in which immunostaining was not feasible due to insufficient tumor sample, the presurgical algorithm was used.

Treatment modalities in the personalized arm were: (i) fgSRLs as monotherapy used for naïve cases which presented a GH_2h_ at sAOT < 2.7 ng/mL or in postsurgical cases when the tumor presented a positive E-cadherin immunoexpression; (ii) combined treatment with fgSRLs and pegvisomant indicated if sAOT showed GH_2h_ > 2.7 ng/mL and %∇GH > 50% as well as a T2 MRI hypointense tumor signal; (iii) those cases identified as a probable non-responders (GH_2h_ > 2.7 ng/mL and %∇GH < 50%) with an MRI that ruled out cavernous sinus invasion or a negative E-cadherin expression in the postsurgical situation were treated with pegvisomant as monotherapy. When discordant results of GH_2h_ and %∇GH were obtained, the result of GH_2h_ prevailed. Regarding those cases in which cavernous sinus invasion was detected by MRI, even if the sAOT predicted a probable non-response to fgSRLs, a combination of fgSRLs and pegvisomant was indicated.

#### Standard treatment algorithm

The standard treatment arm consisted of treatment in concordance with clinical guidelines, starting medical treatment with fgSRLs in all patients at intermediate doses of either octreotide LAR or lanreotide and, in those with failure to control after 6 months full dose of these compounds, to escalate to other treatment modalities upon clinical judgment as recommended by guidelines (surgery, pegvisomant alone or in combination with fgSRLs).

In order to perform a post hoc analysis including the whole cohort, patients in the standard treatment arm also underwent exploration regarding sAOT, T2 MRI tumor signal, and E-cadherin, but their results were not used to define medical therapy in this group.

In both the personalized and the standard treatment arms, either in presurgical cases or in nonsurgically cured patients, if the IGF1-SDS was above 2.5 SDS, doses of the corresponding drugs were increased every 3 months. For combination treatment with fgSRLs and pegvisomant, maximal allowed doses were octreotide LAR 30 mg/monthly and lanreotide 120 mg/monthly in case of inadequate control (IGF1 < 2.5 SDS). After maximal doses of these compounds, pegvisomant was uptitrated at 3 months interval. The use of cabergoline in addition to or in monotherapy was also included in the treatment algorithm if IGF1 was between 2.5 and 3 SDS.

When chiasma compression was detected or when hormonal control was not achieved at the end of the study, surgical treatment was the main recommendation. For postsurgical cases other treatment modalities, either pharmacologic or radiotherapy, were considered upon clinical judgment of their physicians in charge, apart from the study protocols.

### Outcomes

The aim of the study was to assess whether a personalized approach was more effective for achieving hormonal acromegaly control and in a shorter period of time than the classical sequential algorithm. So, the 2 primary endpoints of the study were the percentage of controlled patients at the end of the study (12 months of follow-up) and the time required to achieve disease control in both protocols. Hormonal control of acromegaly was established when IGF1-SDS was normalized. When IGF1-SDS decreased by > 50% over basal value but with no normalization, the patient was considered a partial responder with no control of the disease. In recently diagnosed patients, the minimum follow-up time with no control of the disease despite medical treatment before surgery was scheduled at 6 months. Those patients achieving hormonal control in less than 12 months were considered to be responders; the study was finished for them, and they were eventually referred to surgical treatment if the endocrinologist in charge proposed it.

### Statistical Analysis

The statistical power of the study was calculated considering as significant a 2-sided *P* value of .05 and assuming a beta risk of 0.8. Thus, the minimum number of participants to be included in the trial was 66 subjects, to assess a 30% difference of controlled patients between protocols. Furthermore, the expected loss to follow-up was expected to be 15%. Thus, the final intended recruitment was established to be 76 patients.

Categorical variables were described as number of cases and percentage; and quantitative variables as average ± SDS or median + (p25−p75) or median + (CI). Differences between categorical variables (eg, % of acromegaly comorbidities, % control of the disease) were assessed using the Fisher exact test. Normality of quantitative variables was assessed using the Shapiro-Wilk test. The Student *t* test was performed to analyze differences, or its nonparametric counterpart if the sample distribution was non-normal (Wilcoxon test).

A correlation matrix was constructed with assessment of multiple Spearman's correlation coefficients to identify associations between quantitative variables (age, body mass index [BMI], height, GH_2h_, %∇GH, basal and control GH, IGF1-SDS, tumor diameter and volume, IGF1% variation, and decrease in tumor diameter and volume).

Finally, a survival analysis was performed to analyze time-to-hormone control in both groups. Data were adjusted for age and sex. Results were presented with a 95% CI.

Statistical analyses were performed using the R version 4.2.2 (R Project for Statistical Computing, RRID:SCR_001905). The graphical representation was done using package ggplot 2 (RRID:SCR_014601, Whickham https://CRAN.R-project.org/package=ggplot2) and the *P* values were added using ggpubr package ('ggplot2’ Based Publication Ready Plots, https://CRAN.R-project.org/package=ggpubr). The receiver operating characteristic (ROC) curve was plotted using pROC package (Display and Analyze ROC Curves, https://CRAN.R-project.org/package=pROC).

## Results

### Cohort Description

The final recruited cohort comprised 85 patients; from these, 17 patients were excluded, 13 corresponding to the personalized treatment arm and 4 to the standard treatment arm. Reasons for exclusion were: (i) therapeutic noncompliance (5 patients), (ii) adverse events (4 patients), (iii) withdrawal of consent (2 patients), (iv) surgical treatment performed before obtaining final data of full dose attainment and time treatment response (2 patients), (v) protocol violation (3 patients), and (vi) death before treatment initiation (1 patient). The clinical characteristics of excluded patients are described in the Supplementary Table S1 (36). There were no phenotypical differences between those patients excluded and the rest of the cohort except that dyslipidemia was less prevalent (7% vs 63%, *P* = .02) than in the selected cohort. Thus, 68 patients were finally analyzed and completed the study: 32 patients were in the personalized treatment arm and 36 patients in the standard treatment arm. No clinical differences were found between both groups (Table 2).

**Table 2.** Baseline characteristics of patients with acromegaly by group of treatment

	Personalized treatment (n = 32)	Standard treatment (n = 36)	*P* value
Clinical characteristics			
Gender, ♂/♀	22/10	16/20	.5
Age, years	52 ± 15	56 ± 14	.25
Weight, kg	85 ± 16	82 ± 19	.44
Height, m	1.73 ± 0.09	1.68 ± 0.09	.06
BMI, kg/m^2^	28 ± 4	29 ± 5	.93
Hypertension, %,(n)	34 (11)	36 (13)	1
Type 2 diabetes, % (n)	34 (11)	36 (13)	1
Dyslipidemia, % (n)	34 (11)	42 (15)	.61
Sleep apnea, % (n)	41 (13)	36 (13)	.61
Thyroid nodules, % (n)	34 (11)	42 (15)	.80
Colon polyps, % (n)	13 (4)	17 (6)	.74
Other tumors %(n)	6 (2)	11 (4)	.68
Baseline biochemical and tumor characteristics			
IGF1, SDS	6.1 (4.4-8.1)	5.3 (4.4-6.9)	0.28
GH, ng/mL	4.6 (3.1-16.2)	7.0 (2.8-13.1)	0.87
Largest diameter, mm	17 ± 8	16 ± 8	0.50
Volume, mm^3^	1333 (226-2986)	1590 (168-2787)	0.96
Knosp grade	2.0 ± 1.5	1.85 ± 1.3	0.80
Response predictor factors			
T2-MRI hypointensity, n	18	12	0.20
GH_2h_, ng/dL	1.3 (0.3-2.2)	1.6 (0.4-3.3)	0.34
%∇GH%	−[84 (67-91)]	−[82 (44-90)]	0.48

Abbreviations: BMI, body mass index; GH, growth hormone; GH_2h_, growth hormone value 2 hours after the short acute octreotide test; IGF1, insulin-like growth factor 1; SDS, standard deviation score; %∇GH, percentage GH variation after the short acute octreotide test.

In the personalized treatment arm, 9/32 patients were included after surgical procedure. Of those, 3 subjects were treated according to the presurgical algorithm because it was not possible to obtain sufficient tumor tissue to assess E-cadherin expression. In the standard treatment arm, 10 patients were included after surgery, all of them were treated with fgSRLs as first-line pre-established medical treatment.

### Effectiveness of the Personalized Algorithm and Time to Hormonal Control

**Figure 2.**
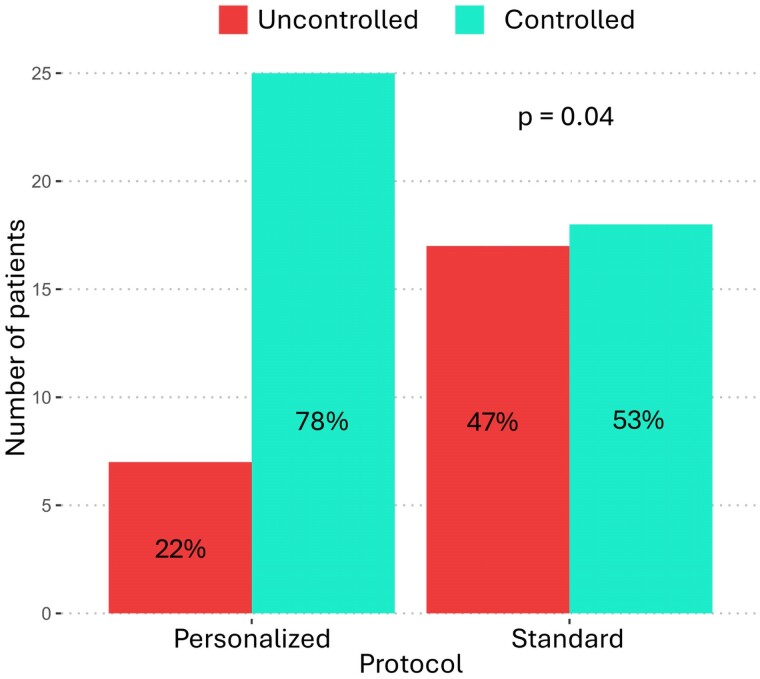
Acromegaly control at the end of the study.

The main outcomes were the percentage of patients controlled and the time spent until achieving control when comparing the personalized approach vs the standard therapy. The study period included a median follow-up of 323 (205-365) days. There were no differences in IGF1 levels at baseline between the 2 groups. At 6 months of follow-up there was already a trend for an enhanced IGF1 control in the personalized protocol: 69% controlled in the personalized vs 47% controlled in the control group (*P* = .07). At the end of the study, the personalized group presented a higher proportion of controlled patients than the standard treatment group (controlled patients 78% vs 53%, *P* = .04, respectively) (Fig. 2).

**Figure 3.**
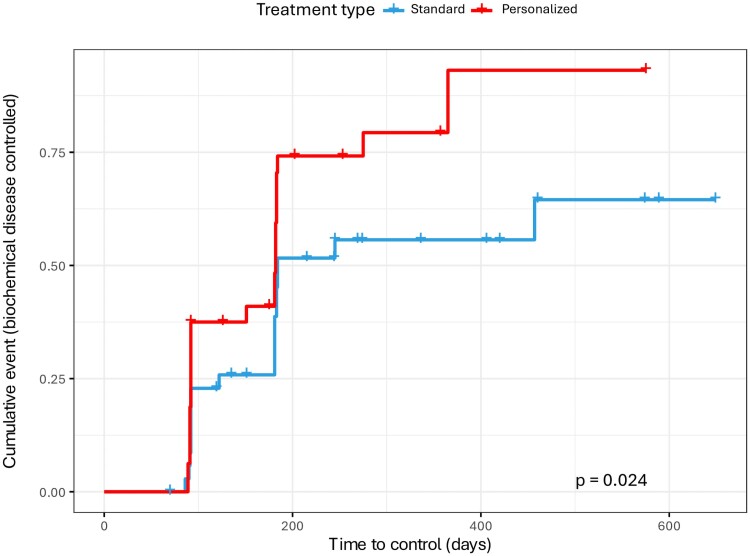
Survival analysis to evaluate differences in time-to-control between groups.

Survival analysis through Kaplan–Meier curves revealed that more patients achieved hormonal control and control was achieved faster in the personalized treatment group (Fig. 3). The hazard ratio for achieving acromegaly control using the personalized protocol was 2.53 (CI, 1.30-4.80) adjusted by age and sex compared to standard treatment. Responder patients to fgSRLs lasted the same time in both groups (150 ± 94 days for personalized group and 158 ± 88 days for standard group; *P* = .1) but differences were found in non-controlled patients from the control group and those predicted as non-responder patients in the personalized treatment arm, in which other treatment than just fgSRLs was used according to the protocol. Assuming a period of 365 days as sufficient to compare both treatment protocols to achieve control, the time-to-control between the predicted non-responders plus the non-responders to fgSRLs from the personalized group and the non-responders from the control group (n = 16 from personalized treatment and n = 17 from standard treatment) was compared. Patients from the personalized arm were controlled faster than the control group (320 [183-365] days vs 365 days, *P* = .01). When we compared all patients from each group, those from personalized treatment were controlled a median of 4 months faster than those from the standard group (182 [92-365] days vs 305 [137-365], *P* = .06 respectively). The comparison of the whole personalized group vs nonresponders from the standard group, showed clearly significant results (182 [92-365] days vs 365 days, *P* < .00001).

### Predictive Ability of the Personalized Algorithm

**Figure 4.**
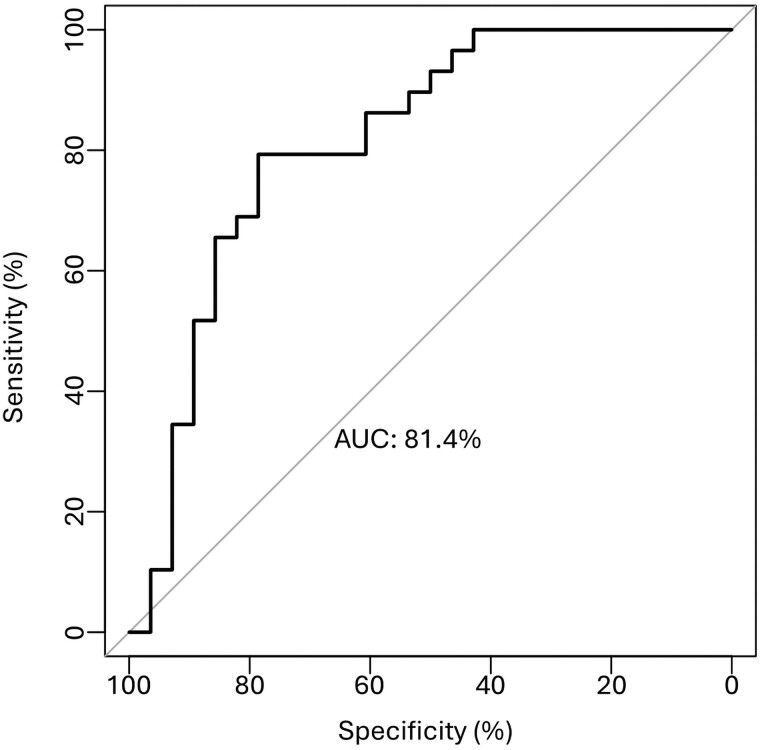
Personalized presurgical algorithm predictive ability to identify first-generation somatostatin receptor ligand (fgSRL) response. Abbreviation: AUC, area under the curve.

ROC curves for the presurgical algorithm indicated a good predictive ability, with an area under the curve (AUC) of 81.4% (CI, 69.8%-93.0%) (Fig. 4).

A simulation of response prediction and control of the disease in the control group was performed according to the data of the sAOT, T2-MRI, and E-cadherin, if available. The personalized algorithms applied to the patients from the control treatment arm, would have foreseen a valid specific positive or negative hormonal control response in the 72% of the group (26 patients out of 36). If the personalized treatment protocol would have been used in the patients included in the control group, 79% of them would have been controlled at the end of the follow-up period, in comparison to what was obtained (53% hormonal control) with the standard treatment, thus superposable to the current 78% achieved in the personalized treatment arm. There were 7% of patients from the control group who would have been overtreated with a combination therapy or pegvisomant as monotherapy, and for whom fgSRLs as monotherapy would have been sufficient to reach hormonal control.

### Factors Associated to Therapeutic Response and Nonresponse Condition

When the cohort was analyzed according to the end of study control achievements, in the personalized arm, the noncontrolled patients (n = 7) were younger (37 ± 7 vs 56 ± 15 years, *P* < .01) and presented a higher BMI (31.0 ± 2.9 vs 27.4 ± 4.5 kg/m^2^, *P* = .04) at baseline. Also, IGF1 and tumor diameter and volume at treatment initiation time were higher in the noncontrolled patients: IGF1-SDS 10.4 (8.0-12.2) vs 5.2 (4.1-6.8) SDS, *P* < .001; diameter 23 ± 9 vs 15 ± 7 mm, *P* = .02; and volume 3478 (2983-6511) vs 947 (187-2088) mm^3^, *P* < .01.

For the standard treatment group, the noncontrolled patients (n = 17) also presented a higher baseline GH (11.1 [4.1-16.1] vs 5.2 [2.3-8.7] ng/mL, *P* = .02) and GH_2h_ (2.2 [1.5-6.0] vs 0.5 [0.2-2.3] ng/mL, *P* = .02), and they had a lower %∇GH (69 [40-83] vs 86 [71-94]%; *P* = 0.02) compared with controlled patients. They also presented a higher tumor diameter (19 ± 7 vs 12 ± 7 mm, *P* < .01) and a higher tumor volume (1939 [1197-3922] vs 571 [37-2110] mm^3^, *P* = .03), with a nonsignificant trend in IGF1 values (5.5 [4.7-8.1] vs 5.04 [3.7-5.9] SDS, *P* = .07).

The personalized treatment group presented no differences in final tumor size compared with the standard treatment group (final diameter = 12 ± 6 vs 10 ± 8 mm, *P* = .44, final volume = 348 [83-1643] vs 472 [43-1415] mm^3^, *P* = .66 respectively). There were no differences in the differential diameter or volume at the end of the study between the 2 groups (differential diameter = 0 –[0-19] vs −8 –[0-17] mm, *P* = .35; differential volume = −14 –[0-61] vs −15 –[0-32] mm^3^, *P* = .67) nor in final diameter or final volume in those patients treated with pegvisomant (either as combination therapy or in monotherapy) vs the rest of the patients (final diameter: 14 ± 5 vs 11 ± 8 mm; *P* = .30; final volume: 750 [246-1539] vs 344 [41-1260] mm^3^). Thus, in no patient treated with pegvisomant was an increase in tumor size detected.

Regarding hormonal control in patients treated with pegvisomant (n = 13; 11 from the personalized treatment group and 2 from the control group), 6 received this drug as monotherapy and 7 in combination with fgSRLs. Eight out of the 11 patients from the personalized treatment arm (72%) achieved normal IGF1 and 3 did not, while any patient from the standard arm achieved biochemical control with pegvisomant added to fgSRLs. Each case is explained accurately in supplementary data (36). All patients required an increase of dosage to 1 to 1.5 mg/kg/w to achieve control.

According to the protocol, dopamine agonists were included in the treatment of 5 patients: 4 belonged to the standard treatment arm and the other one to the personalized treatment arm. Only 2 patients treated with the combination of fgSRLs and cabergoline achieved medical control.

### Correlation Analysis

Initial IGF1-SDS was negatively correlated with age (Rho −0.43, *P* < .001) and positively with initial volume (Rho 0.28, *P* = .03). Furthermore, sAOT GH_2h_ correlated with initial tumor diameter (Rho 0.56, *P* < .001), initial tumor volume (Rho 0.58, *P* < .001), and with several final parameters such as final GH (Rho 0.65, *P* < .001), final IGF1-SDS (Rho 0.36, *P* = .02), final tumor diameter and volume (Rho 0.66, *P* < .001 and (Rho 0.67, *P* < .001 respectively). BMI had a negative correlation with initial and final GH (Rho −0.13, *P* = .01 and Rho −0.39, *P* < .01 respectively). Other expected correlations found were initial tumor diameter and volume with final diameter and volume.

### Adverse Events

Adverse events presented in 9 patients from the personalized group and 9 patients from the standard group. Most of them were mild gastrointestinal and transitory side effects that did not interfere with the study protocol. However, 4 patients were excluded from the study because the adverse events they presented prevented an adequate dose escalation. A detailed list is available in Supplementary Table 2 (36).

## Discussion

The ACROFAST trial marks a significant milestone in the landscape of acromegaly research, offering insights into the feasibility and effectiveness of personalized medical treatment strategies compared to the conventional standard medical therapy approach outlined in most clinical guidelines. Patients with acromegaly have to face the burden of a delayed diagnosis of 10 or more years after disease initiation (37). In addition, in the most recent decades, the recommended drugs for first-line treatment in all clinical guidelines are fgSRLs (1, 2), which have a 50% treatment failure rate (3-6). As currently there are at least 3 additional compounds available (and others will come soon), the “trial-and-error approach” used up until now has to be overcome with a personalized treatment that could guide the decision-making process for acromegaly patients (38, 39). To prove this point, a prospective clinical trial was required to reshape the therapeutic paradigm for acromegaly. As far as we know, ACROFAST is the first prospective trial comparing a personalized treatment vs the standard treatment algorithm recommended in general in clinical guidelines.

In the present study, the primary endpoints selected and the comprehensive assessments employed attempted to provide a holistic evaluation of treatment efficacy and safety. The study was also designed in such a way that its feasibility for daily clinical practice was demonstrable, thus ensuring its general applicability in case of achieving its working hypothesis. Among the different predictive biomarkers used, the sAOT was the capital biomarker in the presurgical algorithm above the T2 MRI intensity, and we were able to confirm its good predictive ability as previously described (11, 16). This test is inexpensive, easy to perform, and fairly interpretable for the clinicians. E-cadherin was used as a biomarker for postoperative cases since it is also a cheap, easy to evaluate, robust, and commonly used biomarker in all pathology clinical laboratories with an even somehow higher predictive ability than SSTR2 (8, 22). Our results also confirm its feasibility, given that only in 6 cases out of 42 it was not possible to determine its expression.

Beside the superior results obtained in the personalized group regarding hormonal control, it has also to be noted that when groups were compared, there were no differences among final tumor size, diameters, and volumes, even if in the personalized group there were patients treated with drugs that did not act on tumor itself as pegvisomant. The fact that almost 80% of patients achieved a controlled IGF1 at the end of the study and earlier in the personalized group is a substantially important achievement, as in the standard treatment group, concordant with the reported efficacy of fgSRLs (6), just 53% of patients achieved hormonal control. On the other hand, the effectiveness of real-world treatment with pegvisomant attained in ACROSTUDY is about 75% (40), which is comparable to the 78% achieved in our study, thus pointing to the important contribution resulting from addition of this drug when required, and ideally at diagnosis if a patient is predicted to be a nonresponder to fgSRLs in monotherapy. The 20% to 25% of patients in which the individualized treatment failed, indicates the necessity of additional effective treatments for predicted nonresponder patients but also, an even more accurate algorithm with more robust predictors.

The correlation matrix of quantitative variables highlights the strong association of the sAOT-GH_2h_ with variables of biochemical control and tumor size (9), as well as reinforcing the already described predictive factors from other retrospective studies, namely, male gender, higher BMI, initial biochemical and imaging data, in relation to biochemical and tumoral responses (5, 15, 16, 41, 42). The ROC curve of the algorithm demonstrated a noteworthy predictive ability of 81.4%, perfectly in line with other described markers (8). When we simulated the application of the algorithm to the standard group, assuming that predicted fgSRL-nonresponsive patients would have been treated with pegvisomant or combination treatment according to the personalized protocol, we obtained a 79% potential controlled patients, with only a 7% risk of overtreatment. This represents a much higher control of the disease than the 53% obtained currently and is concordant with the very similar value of control obtained in the personalized group.

In relation to the treatments given to the personalized group, for the 11 patients in whom pegvisomant was used, a weekly dose of 1 mg/kg was necessary to achieve hormonal control in most cases, while an initial dose of 0.5 mg/kg/week was insufficient. The results of the present study are very concordant, with those reported by the ACROSTUDY, given that a relatively high daily mean absolute dose of 18.9 mg was required to achieve the reported 73% of controlled patients; this means that a standard weekly dose of 2 mg/kg is necessary for a patient weighing 70 kg (43).

Cabergoline was used in 5 patients; however, as described above, hormonal control was only achieved in the 2 cases in which cabergoline was initiated concomitantly with fgSRLs. Thus, the value of cabergoline as a treatment for acromegaly seems limited, unless predictors of response to dopamine agonists would be available to be applied for an individualized treatment.

The one-size-fits-all strategy used to decide the dose of drugs recommended until now as standard therapy clashes with the idea of individualized medicine. On the other hand, it has to be said that if we would have used a higher starting dose of all compounds in the present study, the time-to-control would have been probably shorter. Pasireotide, was not included in the study for instrumental reasons as the number of arms would have increased, but, most importantly, at the time of preparing the trial no predicting factors had been consistently described for being tested in a design like the present one, a situation that is substantially changing nowadays (27). In this regard, a next-step trial on personalized medicine including pasireotide is warranted including imaging (44, 45) and molecular (46) predictors.

The ACROFAST study has several strengths as mentioned above: its accurate methodology and its closeness to clinical practice makes it robust and applicable. The concordance between our results and those of other investigators regarding prevalence of nonresponsiveness to fgSRLs and pegvisomant results denotes its coherency and validity, as well as the demonstrated ability of the proposed algorithm to select the more aggressive tumors. And finally, strengths include the consistency and homogeneity of the groups as well as the robustness of the biomarkers used for identifying responsiveness to fgSRLs, with sufficient statistical power to demonstrate our primary endpoint with a relatively low number of participants. However, the ACROFAST study has also some limitations: although designed as prospective, due to instrumental reasons, an individual randomization was not possible but comparability between patients among the different centers did not show differences; tumors from the patients of the standard treatment group may have been more aggressive—but this does not seem the case in our cohort, thus ruling out this to explain the superiority of the personalized treatment. Also, postoperative cases could eventually have interfered with the results, but we do not consider this probable because there was a very similar number of postoperative patients included in both groups. There were more patients excluded from the personalized group than from the standard treatment one. The exclusion of patients was determined by the external independent committee who were blinded for the patient arm assignment, to avoid any bias, so we believe that we should not underestimate the results of this trial for this reason. The low number of cases and the short period of time using pegvisomant in the standard treatment group as second-line treatment compared with the patients treated with this drug in the personalized treatment group may also be seen as a weakness of the design that could explain the differences on the control of the disease. Interestingly, differences were already almost statistically significant at the 6-month follow-up visit, when fgSRLs were used at maximal doses in the standard group vs the different strategies adopted in the personalized group. We assume that, eventually, a longer period of time with other treatments in the standard arm would have achieved similar rate of control than in the personalized arm, but obviously consuming more time, which was in fact one of the working hypothesis of the present study. Moreover, in the personalized group there were only 2 additional patients who benefited from the intensification with pegvisomant as second-line treatment so, the relevance of the intensification strategy is relatively small although this could be also another limitation of the study. Finally, the study was performed while in the very middle of the COVID-19 pandemic, which obviously made everything more complicated; however, we were able to manage, and we do not believe that it influenced the results obtained.

Thus, concluding, the results of the present study, the very first ever performed and without any pharmaceutical financial bias, comparing standard therapy with a personalized protocol, indicate a superior and faster hormonal control and the consequent improved symptomatic relief in the personalized treatment arm. The ACROFAST trial represents a pioneering effort to redefine the therapeutic management of acromegaly through personalized medical treatment. Its implications on clinical practice and guideline recommendations are eagerly anticipated, with the potential to clearly improve the clinical care of individuals with acromegaly.

## Data Availability

Datasets generated during and/or analyzed during the current study are not publicly available but are available from the corresponding author on reasonable request.

## Trial Registration Number

MPD-SOM-2019-01.

## Abbreviations

fgSRLs: first-generation somatostatin receptor ligands
GH: growth hormone
MRI: magnetic resonance imaging
ROC: receiver operating characteristic
sAOT: short acute octreotide test
SSTR2: somatostatin receptor 2
