# Correction to: “Personalized Medicine in Acromegaly: The ACROFAST Study”

**DOI:** 10.1210/clinem/dgae665

**Published:** 2024-10-03

In the above-named article by Marques-Pamies M, Gil J, Sampedro-Nuñez M, Valassi E, Biagetti B, Giménez-Palop O, Hernández M, Martínez S, Carrato C, Villar-Taibo R, Araujo-Castro M, Blanco C, Simón-Muela I, Simó-Servat A, Xifra G, Vázquez F, Pavón I, Rosado JA, García-Centeno R, Zavala R, Hanzu FA, Mora M, Aulinas A, Vilarrasa N, Librizzi S, Calatayud M, de Miguel P, Alvarez-Escola C, Picó A, Salinas I, Fajardo-Montañana C, Cámara R, Bernabéu I, Jordà M, Webb SM, Marazuela M, and Puig-Domingo M (*J Clin Endo Metab*. 2024; doi: 10.1210/clinem/dgae444), there was a typographical error in Figure 1.

In the originally published article, in Figure 1, the word “Hypointensity” that is above the down-arrow pointing toward “FgSRLs + Pegvisomant (N 5),” has been corrected to read “Hyperintensity.”

**Original** Figure 1:

**Figure:**
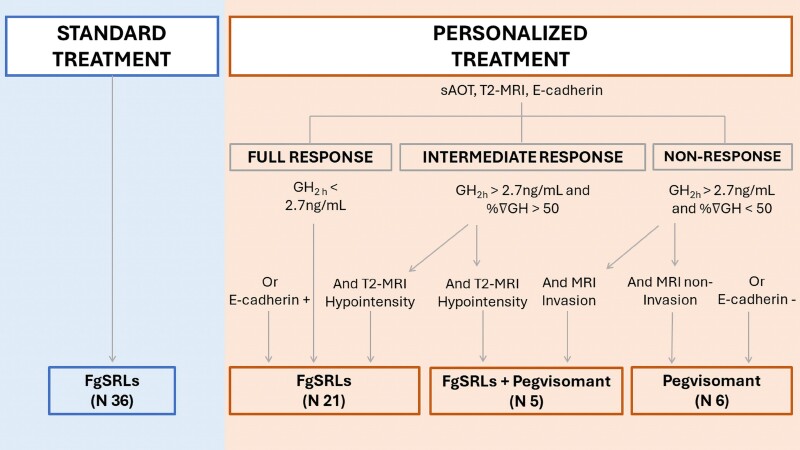


**Corrected** Figure 1:

**Figure:**
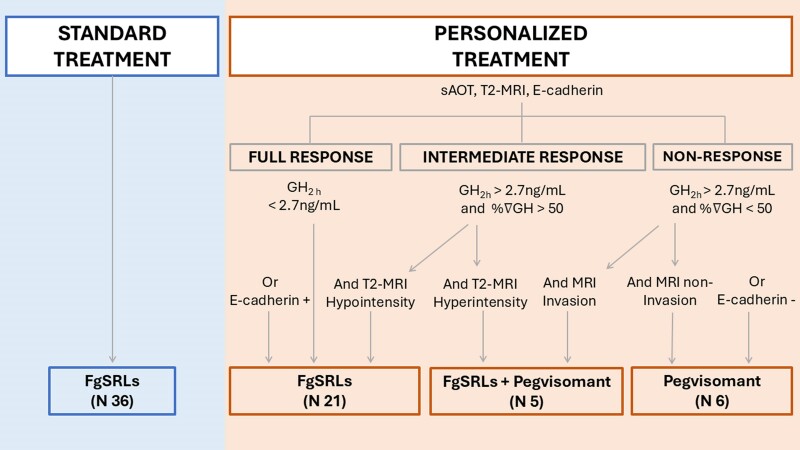


The article has been corrected online.

doi: 10.1210/clinem/dgae444

